# Autoimmune Features of Post-COVID-19 Vaccination Syndrome and Their Impacts on the Renin–Angiotensin System

**DOI:** 10.3390/vaccines14040354

**Published:** 2026-04-16

**Authors:** Paolo Bellavite, Giuseppe Di Fede, Mauro Mantovani, Elisabetta Zanolin

**Affiliations:** 1 Independent Researcher, 37121 Verona, Italy; 2 Istituto di Medicina Biologica, 20129 Milano, Italy; mauromantovabi72@gmail.com; 3 Unit of Epidemiology & Statistical Medicine, Department of Diagnostics and Public Health, University of Verona, 37134 Verona, Italy; elisabetta.zanolin@univr.it

**Keywords:** COVID-19 vaccination, post-acute COVID-19 syndrome, post-acute COVID-19 vaccination syndrome, spike protein, angiotensin converting enzyme 2, renin angiotensin system, autoantibodies, MAS1 receptor, idiotype network, anti-idiotype

## Abstract

One of the most critical aspects of post-acute COVID-19 syndrome (PACS) and post-acute COVID-19 vaccination syndrome (PACVS) is the presence of autoantibodies. These autoantibodies are directed against various receptors in the autonomic and cardiovascular systems, including those targeting proteins of the renin–angiotensin system (RAS). The RAS plays a central role in regulating vascular homeostasis, inflammation, and endothelial function. During SARS-CoV-2 infection, the interaction of the spike (S) protein with angiotensin-converting enzyme 2 (ACE2) can alter the balance of the RAS, favoring an imbalance towards the ACE/Angiotensin II/AT1R axis, known for its pro-inflammatory, pro-thrombotic, and vasoconstrictive properties. Similar pathological mechanisms also come into play in response to vaccinations that use the S protein as an antigen. Studies conducted by other groups and us on patients with PACS and PACVS have revealed the presence of autoantibodies directed against these RAS components and the mechanisms by which these antibodies can worsen the clinical situation. In particular, anti-ACE2, presumably formed by the anti-idiotype network or molecular mimicry, is correlated with PACVS symptoms in many patients. Furthermore, the presence of anti-MAS1 antibodies can reduce the efficiency of the ACE2/Angiotensin-(1–7)/MAS1 axis, which normally acts as a counter-regulator. Considering this evidence, an analysis of RAS molecules and the autoantibodies implicated in reactions to them may be useful for evaluating a state of persistent dysregulation associated with post-vaccination symptoms such as asthenia, headache, skin edema and bruising, cardiovascular alterations, and neurovegetative manifestations. Finally, we offer insights into diagnosing these multifaceted syndromes and working hypotheses to guide research into possible therapeutic approaches.

## 1. Introduction

It has long been known that COVID-19 can lead to complications that prolong the course of the disease and make its treatment more difficult (“long COVID” or “post-acute COVID-19 syndrome”—PACS), especially in relation to its severity [1,2,3,4]. The prevalence of this condition varies widely across the literature depending on the definition used, the virus variant, the symptoms considered, and their duration (weeks or months after infection). In the UK, long COVID has been experienced by 4.5% and 10.8% of omicron and delta cases, respectively [5]. A deterioration in health, with various symptoms of neurological and/or cardiac origin, is frequent after overcoming the acute phase of COVID-19, and it has been hypothesized that the presence of autoantibodies directed against G protein-coupled receptors (GPCRs) may be involved [6].

A large body of literature has highlighted how even COVID-19 vaccines can cause adverse reactions that last over time (months or years), even once the acute phase has passed [7,8,9,10,11], often referred to as Post-Acute COVID-19 Vaccination Syndrome (PACVS) [12,13,14,15,16,17,18]. A first scientific report of many patients complaining of long-term side effects after COVID-19 vaccination—primarily brain fog, headaches, and blood pressure fluctuations—appeared in Science on 20 January 2022 [19]. The same report noted that the NIH significantly delayed acknowledging the problem, which patients had been complaining about since spring 2021, and that this silence was “distressing”. The authors state that several patients reported that doctors were unsure of how to manage the disease and sometimes claimed that the symptoms were imaginary.

PACVS is a highly heterogeneous syndrome that can present various physical symptoms affecting the entire body, such as general fatigue and hypertension, various organ systems with muscle and joint pain, numbness of the extremities, dyspnea, tachycardia, as well as many neurological and neuropsychiatric symptoms such as insomnia, anxiety, and dizziness.

Many signs and symptoms of PACVS, which characterize a disturbance of the autonomic and immunoendocrine responses to vaccination, are similar to those of PACS, suggesting, at least, a partially shared pathogenesis [10,14,20]. Autoimmune mechanisms have also been implicated in the adverse effects of vaccination [21,22,23,24,25], but it is unclear which types of antigens and antibodies are involved. Some authors have found increased levels of autoantibodies against angiotensin-converting enzyme 2 (ACE2, the main receptor of SARS-CoV-2) and a series of GPCRs such as MAS1 or adrenergic and cholinergic receptors, as well as towards Angiotensin II type 1 receptor (AT1R), Endothelin A receptor (ETAR), the chemokine receptor CXCR3, stabilin-1 (Stab1), Fibroblast growth factor receptor 3 (FGFR3), and Platelet factor 4 (PF4) [26,27,28,29,30]. The suspicion that these are immune-mediated disorders also arises from their similarity with symptoms of postural orthostatic tachycardia (POTS) and chronic fatigue syndrome (CFS), both of which have been proposed to be autoimmune. Specifically, elevated levels of autoantibodies directed against cardiac proteins, acetylcholine receptors, β-adrenergic receptors, and muscarinic receptors type 2 and 3 have been detected in POTS [31].

The distinction between PACS and PACVS is difficult because the symptoms are similar, and, in both cases, the pathophysiological mechanisms are largely due to a distorted reaction to S proteins of the SARS-CoV-2 virus or induced by vaccines [10,24,25,32,33,34].

Overall, PACVS remains a poorly understood condition that requires further research to clarify its prevalence, prognosis, risk factors, and treatments. This is due to three main reasons: (a) the long-term consequences of vaccinations are systemic and affect multiple organs; (b) autoimmune diseases are always influenced by individual susceptibilities, and therefore, the same cause can develop different abnormalities and take on different phenotypic aspects in different individuals; and (c) the pathophysiological and immunological mechanisms of the response to the vaccine and virus overlap in many respects, making it difficult to distinguish the two situations when most vaccinated patients have also contracted the viral disease.

In some cases, PACVS presents signs and symptoms typical of diseases already recognized as established diagnoses, such as small fiber neuropathy (SFN) [22,30,35,36], POTS [18,37], CFS [11], or as long-term sequelae of heart disease [38,39]. In other cases, the symptoms are much more subtle and variable, including manifestations ranging from paraesthesia to visual disturbances, dizziness to neuropsychiatric disorders, and dermatitis to coagulopathy. Skin inflammatory symptoms have also been reported as long-term sequelae of COVID-19 vaccines [40,41,42,43,44].

The main signs and symptoms of PACVS, as reported by recent papers on this subject [10,11,12,15,45,46,47,48], are reported in Table 1.

The pathogenesis of PACVS encompasses many aspects involving genetic, metabolic, immunological, and mental factors. In articles dedicated to post-COVID-19 syndrome and PACVS, other authors have highlighted how S proteins can damage mitochondria and cause energy metabolism disorders, which could explain symptoms such as chronic fatigue and brain fog [16,47,49,50]. However, this does not explain symptoms more closely related to inflammatory phenomena, which are more likely associated with immune system reactions.

A particular difficulty for those affected by the long-term consequences of COVID-19 vaccines is that PACVS is not yet codified as a recognized disease by public health systems. Many common symptoms of PACVS, considered separately, have been recorded by pharmacovigilance systems as adverse reactions, but health authorities have so far failed to address this clinical entity appropriately [51]. The fundamental problem is that there is no consensus on which specific clinical and diagnostic correlates exist. Consequently, determining the correlation between the disease and vaccination becomes even more challenging. This also depends on the fact that adverse reactions to vaccines are typically recorded in the first few days or, at most, the first few weeks following inoculation, while the duration of symptoms is poorly monitored by post-authorization pharmacovigilance.

The prevalence of PACVS among vaccinated people has been estimated at around 0.02% [12], but no consistent epidemiological data have been reported. However, active pharmacovigilance studies in young or working-age people indicate a prevalence of 0.9% [51]. In a study among healthcare workers in the Czech Republic [52], 1.4% of participants reported adverse effects after the mRNA vaccine (Pfizer) lasting more than a month, although the symptoms that lasted that long were not described. In other active pharmacovigilance studies, general symptoms such as chronic fatigue, headache, and joint and muscle pain lasted more than a month in 0.4% [53] or 0.2% of participants [54]. These different estimates likely depend on the fact that it is not yet clear which criteria or markers can be used to diagnose PACVS and distinguish it from other post-vaccination pathologies with a semi-acute or chronic course, such as specific organic diseases (hepatitis, myocarditis, nephritis, etc.).

In this review, we contribute to understanding the pathogenesis of PACVS by addressing autoimmunity and analyzing the immunopathological mechanisms identified to date. We begin by summarizing some laboratory evidence from our observational studies and clinical cases, and then examine aspects related to the classic anti-idiotype mechanisms and the molecular mimicry between the vaccine-derived S protein and molecules present in the human body. We concentrate our attention particularly on autoimmunity to ACE2 and MAS1, two key components of the renin–angiotensin system (RAS). Finally, we discuss the complex and multifaceted diagnostic aspects of the syndrome and formulate rational hypotheses about potential therapies, which could guide future controlled clinical research in this field.

## 2. Observational Study and Case Reports

Our research group, featuring a collaboration between the Institute of Biological Medicine in Milan and professors at the University of Verona, studied a patient with severe PACVS, which initially caused thrombosis and then dysautonomic syndrome [45]. When studying this patient, we observed a significant increase in anti-GPCR antibodies, which then decreased with two therapeutic plasmapheresis treatments. After an initial experience with positive outcomes, we continued to see patients with long-term consequences of vaccination. From our case series, we extracted the clinical history and laboratory data of 17 individuals (13 women and 4 men; mean age, 44) who had not been previously infected with COVID-19 and presented with symptoms compatible with PACVS [46]. These individuals had received multiple doses (min 1, max 3) of adenoviral vector (*n* = 2) or mRNA (*n* = 15) vaccines, and their median observation period after vaccination was 20 months (min 4, max 32). Patients were healthy before vaccination, and all had anti-S but not anti-N IgG antibodies. Therefore, it is almost certain that their disease was due to vaccination. Since then, our clinical knowledge has expanded with additional cases, and we have gained greater experience in diagnostic approaches and therapies, though these remain preliminary and observational.

The PACVS cases we studied allowed us to observe the main laboratory and immunopathological alterations. A summary of this analysis is provided here, along with a discussion of the primary immunological disorder observed.

**Figure 1:**
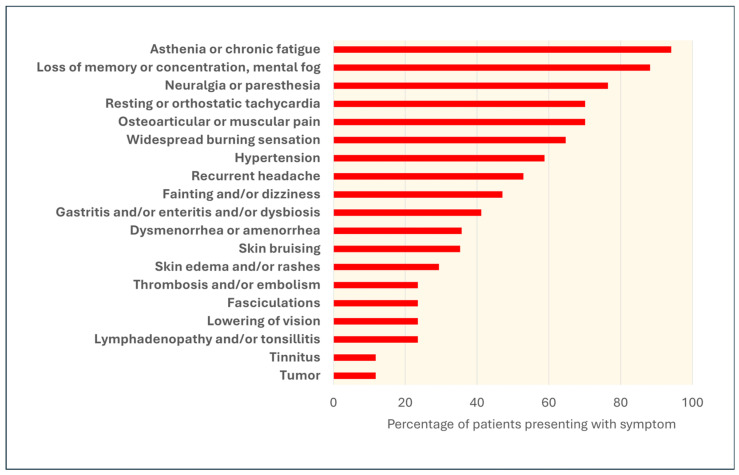
Percentage frequency of specific symptoms of 17 patients with PACVS. Data from our case study [46].

Our patients’ symptoms are shown in Figure 1, from the most prevalent (top rows) to the rarest (bottom). All patients had several of the symptoms listed here, with a mean of 9.1 ± 3.5 (SD) symptoms per patient (maximum: 16, minimum: 4).

The symptoms reported by almost all patients included severe fatigue, loss of concentration, amnesia, a sensation described as “brain fog,” and neurological symptoms such as neuralgia or diffuse paresthesia, often described as an internal “burning” sensation. Symptoms of cardiovascular dysautonomia, such as resting and/or orthostatic tachycardia and hypertension, were very common. Arthralgia and/or muscle pain were reported by approximately 70% of patients. Headache, fainting or dizziness, and visual disturbances were also common. Approximately half of the patients had skin conditions, often recurrent, such as erythematous and/or edematous rashes and ecchymoses. Dysmenorrhea or amenorrhea was frequently reported in female patients. It should be noted that the reported symptoms were severe and persistent or presented as recurrent crises and, in all cases, significantly impacted quality of life.

Among the 17 patients, 13 had Interleukin 1-beta levels above the normal limit, and 11 also had increased Interleukin 8 levels, consistent with the results reported by others. In 16 patients, T regulatory cell (Treg) levels were below the lower limit of normality, consistent with an autoimmune mechanism [55] and with the findings in severe cases of COVID-19 [56,57]. Among 15 patients in whom plasma angiotensin-(1–7) [ang-(1–7)] concentration was measured, 10 (66.6%) presented levels above the normal limit (226 ng/mL).

**Figure 2:**
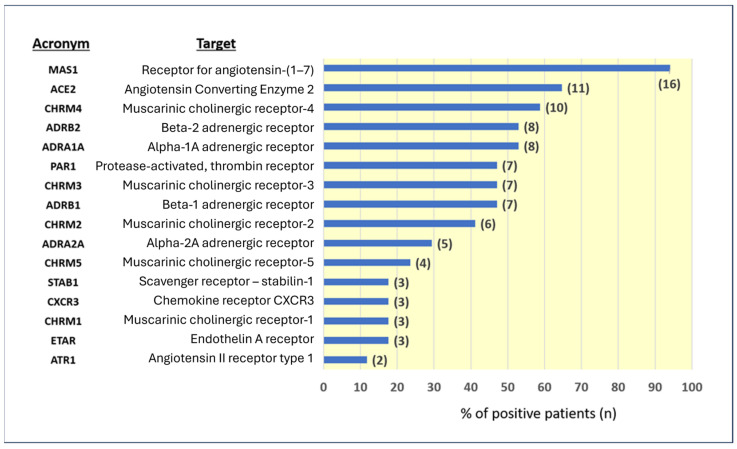
Percentage (number) of autoantibodies positive in the serum of a group of patients with PACVS symptoms. The panel of 16 antibodies was tested from frozen serum using commercial ELISA kits by Prof. Dr. Kai Schulze-Forster and Dr. Harald Heidecke at CellTrend GmbH–ImBio technologiepark, Luckenwalde, according to the manufacturer’s instructions (https://www.celltrend.de/). The test was scored as positive if the antibody level was above the positivity threshold established specifically for each antibody, using a value determined in healthy subjects by the laboratory that performed the assays [46,58].

Antinuclear antibodies (ANAs), anti-neutrophil cytoplasmic antibodies (ANCAs), extractable antinuclear antibodies (ENAs), and anti-cardiac muscle antibodies were negative in all cases tested. Given the negativity of more conventionally recognized autoantibodies and suspicion of the disease’s autoimmune nature, we initiated more specialized antibody tests that are not commonly performed. These tests include a panel of 16 antibodies against RAS-related proteins (ACE2 and MAS1) and other GPCRs. The results in our patient series are shown in Figure 2.

In most patients, antibodies against MAS1, ACE2, muscarinic cholinergic receptor-4, beta-2 adrenergic receptor, and alpha-1A adrenergic receptor gave positive scores. It was, therefore, interesting to see whether positivity to any of these antibodies correlated with specific symptoms. For most antibodies, no correlation with symptoms was found, which suggests that they are not involved in the expression of pathology or represent an internal immune system adjustment process without direct organic implications.

**Figure 3:**
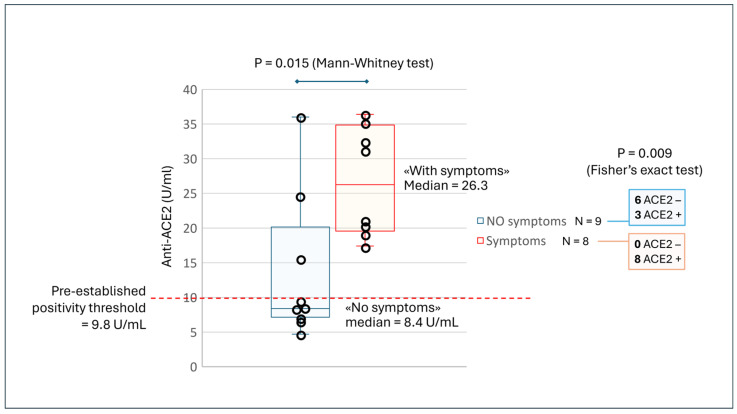
Box-and-whisker plot and association (assessed via Mann–Whitney test) of anti-ACE2 antibody levels in subjects with or without symptoms of bruising, skin edema, or rashes. Right part of figure: association (assessed via Fisher’s exact test) between the presence of cited symptoms and ACE2 positivity. Figure based on our data from 17 patients with PACVS [46].

Although the sample was small, we found a statistically significant correlation between anti-ACE2 positivity and symptoms such as bruising, skin edema, or rashes (Figure 3).

Across the 17 patients, 9 without symptoms had a median antibody concentration of 8.4 U/mL (interquartile range = 7.5–15.6), while those with symptoms had a median of 26.3 (interquartile range = 19.8–34.0; Mann–Whitney test: *p* = 0.015). Moreover, of the 11 ACE2-positive patients, 8 had such symptoms, while among the 6 ACE2-negative patients, none had such symptoms (Fisher’s exact test: *p* = 0.009).

Furthermore, a more detailed analysis of the correlations between antibodies and symptoms presented by patients highlighted that cases positive for ACE2 antibodies had a higher prevalence of hypertension, headache, gastritis, bruising, skin edema, rashes, thrombosis, and decreased vision compared to cases negative for the same antibodies (*p* < 0.001) [46].

In our patients, we observed an increase in anti-MAS1 antibodies and, at the same time, an increase in ang-(1–7) in two-thirds of them. Almost all patients had anti-MAS1 levels above the normal threshold, and measuring these antibodies clearly distinguished patients with symptoms such as widespread burning sensations from those without these symptoms. The median serum level of MAS1 antibodies in 5 patients without these symptoms was 30.9 U/mL (range 28.7–44.6), while that in 12 patients with these symptoms was 51.6 U/mL (interquartile range 45.1–53.6) (Mann–Whitney test: *p* < 0.009).

In a recent case observed in our outpatient clinic (not included in the previous study), the increase in anti-MAS1 was particularly marked and represented, by far, the greatest deviation when compared to other antibodies of the autoimmune panel used. A 55-year-old woman who was affected by post-COVID-19 vaccination illness primarily experienced arthralgia, skin erythema, widespread burning paresthesia, and recurrent hypertensive crises. In this patient, most antibodies were negative: anti-α-1-adrenergic receptor and anti-β-2-adrenergic receptor were just above normal, while anti-MAS1 was well above the threshold of pathology (25.0 U/mL) at 94.5 U/mL. ANA, ENA, and ANCA assays yielded negative results. Details of this case are reported in the Supplementary File S1.

Another chief aspect is that recent tests conducted in our laboratory revealed high levels of anti-SARS-CoV-2 S-RBD IgG and anti-SARS-CoV2 Nucleocapsid IgG and IgM antibodies. These data suggest that the immune system remains stimulated, which may explain the persistence of the autoimmune disorder. Clearly, the presence of anti-nucleocapsid antibodies also suggests a viral infection, but the clinical history indicates that the adverse effects appeared a few days after vaccination and before first infection with SARS-CoV-2, occurring on and documented via PCR on 24 December 2021. It is important to consider that, in relation to the mechanisms highlighted here, once the disorder is triggered by the vaccine, any new encounter with viral antigens, even without causing severe disease, can theoretically rekindle autoimmunity.

Genetic analysis has documented the presence of both C677T and A1298C mutations/polymorphisms in the methylenetetrahydrofolate reductase (MTHFR) gene in the heterozygous state. This may suggest the existence of a genetic predisposition that caused the disease after the vaccine stimulus, which served as a trigger. Indeed, others have reported an MTHFR mutation in patients with severe COVID-19 [59,60,61], COVID-19 vaccine adverse events [62,63,64], and other autoimmune diseases [65].

Clearly, a single case cannot demonstrate that the cause of PACVS in this patient is precisely an excess of anti-MAS1. However, it highlights the phenomenon’s significance, as the criteria that must be satisfied to establish a causal link include, in addition to a compatible time frame and the absence of other alternative explanations for the pathology, also the biological plausibility and the presence of similar cases described in the literature [66,67]. In the next sections, we will examine how RAS disorders induced by the presence of anti-ACE2 and anti-MAS1 autoantibodies could explain at least some of the symptoms reported by patients with PACS and PACVS.

## 3. Immunopathological Aspects of PACVS

The first new-onset autoimmune phenomena reported after COVID-19 vaccination were immune thrombotic thrombocytopenia, liver disease, Guillain–Barré syndrome, IgA nephropathy, rheumatoid arthritis, alopecia areata, psoriasis, hypertensive crisis, myocarditis and pericarditis, type 1 diabetes mellitus, and hepatitis [68,69,70,71,72,73,74,75,76]. Vaccination against COVID-19 has already been reported to increase the risk of developing kidney disease [77], autoimmune liver disease [78], systemic lupus erythematosus [79], and a booster dose of the vaccine has been associated with an increased risk of some autoimmune diseases, including alopecia areata, psoriasis, and rheumatoid arthritis [73]. Flares of autoimmune rheumatic diseases occur in nearly 1 in 10 patients following COVID-19 vaccination [80].

Regarding neurological sequelae, both following COVID-19 and its vaccination, complications such as mental status and language disorders, behavioral changes, movement difficulties, and seizures have been described [81,82,83]. As also observed in our cohort (Figure 1), among the chronic sequelae of vaccines, dermatological problems related to inflammation, particularly urticarial symptoms, have often been reported [40,41,42,43,44,84,85]. In a cohort of 159 females and 32 males with PACVS, 19.37% had symptoms superimposable to those of mast-cell activation syndrome (MCAS) [12], prominent symptoms of which are urticaria, angioedema, and flushing [86].

More recently, it has been understood that many autoimmune manifestations do not affect a single organ but are systemic in nature, with notable variations between different individuals, which has led to the emergence of a new autoimmune-based syndrome called PACVS. This is similar to what has been observed with the after-effects of COVID-19, which are grouped under the definition of PACS.

It is important to note that SARS-CoV-2 virus infection is also associated with an increased risk of autoimmune diseases such as spondylarthritis, rheumatoid arthritis, psoriasis, pemphigoid, Graves’ disease, anti-phospholipid antibody syndrome, immune-mediated thrombocytopenia, multiple sclerosis, and vasculitis [79,87]. Therefore, vaccination can cause autoimmunity but may also indirectly protect against autoimmunity caused by the virus. Balancing the benefits and risks of vaccines is a highly complex assessment that goes beyond the scope of this review, as it requires knowledge of individual susceptibility and, above all, the epidemiological risk of the disease and its severity at different stages of life.

The mechanisms of post-vaccination pathology vary due to the complex composition of lipid nanoparticles or adenoviral vaccines. However, among the primary sources of acute or chronic adverse reactions is the S protein [18,24,32,34,88,89]. Following production by vaccine-transfected cells, this protein is expressed on the cell membrane or released into the lymph node environment, and may also enter the blood and be distributed to various tissues. The S protein has very similar, though not identical, characteristics to those of the virus [24]; it somewhat mimics the pathogenic action of the virus, interacting with ACE2 receptors and triggering dysregulated immune responses. The main mechanisms explaining the onset of autoimmunity are the following:

1. The adjuvant effect of lipid nanoparticles [90] or the presence of impurities [91,92,93], which are possibly batch-dependent [94,95];
2. Cell synthesis of anomalous frame-shifted recombinant S proteins or their fragments [96,97];
3. Molecular mimicry, probably due to the similarity of the S protein with human cell components [24,28,76,98,99,100,101];
4. Idiotype network [102,103] and epitope spreading [104];
5. Genetic background, e.g., HLA haplotype [100], MTHFR [62,63], female gender [105,106].

**Figure 4:**
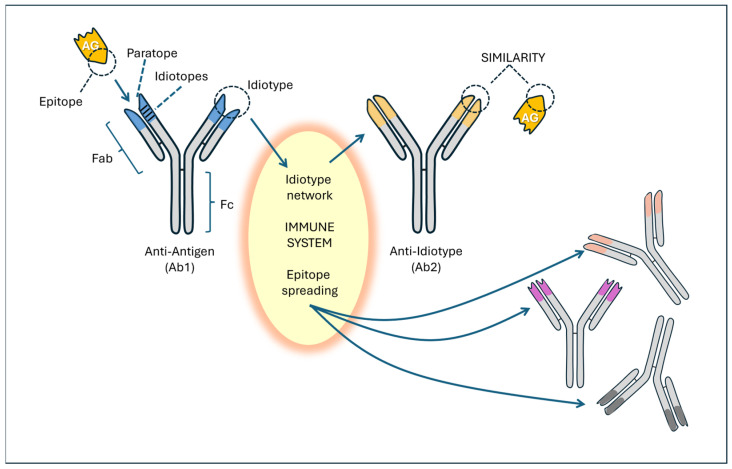
Schematic illustration of the formation of anti-idiotypic antibodies. The epitope (or antigenic determinant) is the small portion of the antigen that binds the specific antibody. The paratope is the specific region of an antibody (Ab1) that recognizes and binds to the antigen epitope, functioning as a “key” that fits into the antigen’s “lock”. The term idiotype denotes an entire collection of idiotopes and paratopes present in a single immunoglobulin molecule that develops due to somatic mutations in B lymphocytes [107]. Paratopes, representing parts of “new” proteins, have the potential to induce anti-idiotype antibodies (Ab2), which can be considered an “internal image” of the original external antigen. In the case of COVID-19 vaccines, some Ab2 clones may have parts of the molecule similar to the S protein and, consequently, could also bind to its target, ACE2. Epitope spreading is defined as the diversification of epitope specificity from the initial focused and dominant epitope-specific immune response, directed against a self- or foreign protein, to subdominant and/or cryptic epitopes on that protein (intramolecular spreading) or other similar proteins (intermolecular spreading) [109].

Considering the importance of ACE2 as SARS-CoV-2’s major receptor and its enzymatic function within the RAS, we [24,46] and others [102,107,108] developed a hypothesis according to which anti-ACE2 antibodies would be formed through an anti-idiotype immunologic mechanism (Figure 4).

The idiotypic network represents one of the most important immunological theories, alongside clonal selection (which explains the specificity of the immune response) and clonal deletion (which explains immune tolerance). This concept, developed by Niels Jerne [110], who described it with extraordinary clarity at the end of the last century, is based on the idea that the idiotype (from the Greek ἴδιος/’idios/, “own”)—that is, the region of an antibody that binds to the antigen—can be recognized by the immune system as a potentially foreign structure and may, consequently, become the target of another antibody, called an anti-idiotype.

Idiotypes can be considered foreign and, therefore, immunogenic proteins. The anti-idiotype antibody (Ab2) binds to the idiotypic variable domains of an antibody. The paratope of the Ab2 antibody mimics the original antigen as if it were a complete internal image of the antigen and can display a functional activity that resembles the natural physiological activity of the antigen. According to the immune network theory [110], a network of different clones between anti-idiotypic antibodies (Ab2, Ab3, Ab4, etc.) is developed [103,111,112]. This network can generate a large series of anti-idiotypic receptors and antibodies, which broadens the repertoire of epitopes [76,113,114].

Although the idiotypic mechanism is the most likely, a classic molecular mimicry mechanism may also be involved in autoimmunity. Indeed, the S protein shares significant homology with various human proteins or peptides [98,99,115,116,117]. Many human proteins have been indicated as being involved in the mimicry phenomenon because they have peptides in common with the S protein [99]. These authors have underlined the cross-reactivity with adiponectin, with the adenosine A2b receptor, with the macrophage C163A receptor, with the transcription factor CREB1, which controls the expression of NF-κB, and with IL-10. A large number of shared pentapeptides between the S protein and H. sapiens proteins have also been identified, among which 39 proteins were linked to axon and myelin homeostasis [118]. In particular, some proteins, such as phosphacan, attractin, teneurin-4, and myelin-associated glycoprotein, were identified as susceptible targets of B and T cells. According to Nunez-Castilla et al. [119], a TQLPP (Threonine–Glutamine–Leucine–Proline–Proline) motif in S proteins shares similar antibody binding properties with thrombopoietin and may induce thrombocytopenia. Another motif, ELDKY (Glutammic Ac.–Leucine–Aspartic Ac.–Lysine–Tyrosine), is shared by multiple human proteins, such as protein kinase G1, involved in platelet activation, and tropomyosin, linked to cardiac disease.

Mizuno et al. [120] evaluated molecular mimicry both bioinformatically and in vitro, and identified an antibody with high affinity for the spike protein cross-reacting with an EPLDVL motif (Glutammic Ac.–Proline–Leucine–Aspartic Ac.–Lysine–Valine–Lysine) present in several human proteins expressed mainly in the small intestine, ovaries, and stomach. A significant proportion of patients infected with SARS-CoV-2 have developed autoantibodies against angiotensin II, a phenomenon that could explain the dysregulation of blood pressure and a greater severity of the disease [121]. These autoantibodies would be formed by a cross-reaction between the receptor-binding domain of protein S and angiotensin II, thus suggesting a certain epitopic mimicry between the two proteins, both being capable of binding to ACE2. Based on these findings, re-evaluation of the vaccine composition and exclusion of cross-reacting peptides from vaccine epitopes were recommended to prevent possible autoimmune reactions [118,120].

A recent analysis showed that the S protein shares structural elements with ACE2, with alignments that encapsulate information on the transcripts, hydrophobicity, and secondary structure of the protein (five levels of concordance in total) [122]. Specifically, segments of the SARS-CoV-2 S protein (residues 49–53, 226–230, 418–428, 529–536, 744–774, and 977–980) share structural and chemical similarities with some segments of the ACE2 receptor (residues 137–141, 335–345, 350–352, 463–471, and 545–573). These areas could be parts of functional structural motifs common to both proteins.

Furthermore, a pathogenic role could be played by the lipid nanoparticles that characterize these vaccines, composed of substances such as polyethylene glycol, cationic lipids, phospholipids, and cholesterol, which can act as adjuvants, stimulating the secretion of pro-inflammatory cytokines such as IL-1 beta, IL-8, and reactive oxygen species [123,124].

### 3.1. The Role of ACE2

At the center of this discussion is ACE2, which represents the entry point for both the SARS-CoV-2 virus and the receptor of the vaccine S protein. However, ACE2 is not just a receptor: it also exhibits enzymatic activity, catalyzing the conversion of angiotensin II to ang-(1–7) and playing a role in bradykinin metabolism. This dual function, both receptor and enzymatic, is crucial for multiple physiological processes. From a receptor perspective, ACE2 facilitates viral entry into cells; from an enzymatic perspective, its inhibition leads to the accumulation of angiotensin II, with consequent pro-hypertensive, pro-coagulant, and pro-inflammatory effects, as well as increased oxidative stress and bradykinin-mediated pain. Furthermore, the involvement of the ACE2 receptor in platelet activation leads to increased platelet aggregation, facilitating thrombosis.

**Figure 5:**
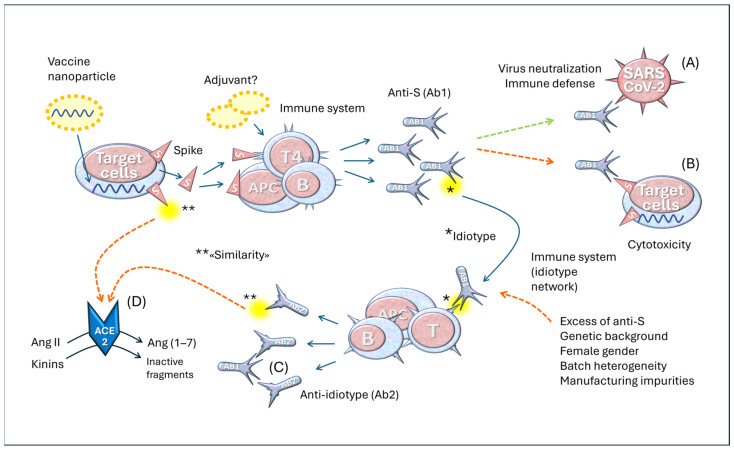
Simplified conceptual model of the formation of anti-S and anti-ACE2 antibodies following COVID-19 vaccination. The upper part illustrates the synthesis of anti-S antibodies (Ab1) and their useful anti-virus (A) or harmful and cytotoxic (B) functions. The lower part illustrates the synthesis of anti-idiotype antibodies (Ab2), which act by maintaining immune homeostasis (C) or by interacting with ACE2, thereby disrupting RAS homeostasis (D). This figure does not illustrate the presence of soluble serum ACE2 receptors (sACE2), which adds another layer of complexity to the pathophysiology. Solid arrows: changes in the immune system and production of antibodies; green dashed arrow: physiological effects; red dashed arrows: pathological effects. * Molecular complementarity of idiotypes. ** Molecular similarity between S protein and Ab2.

In the best-case scenario, as illustrated in Figure 5 (upper part), the vaccine induces the production of the S protein, which stimulates the immune system to generate specific antibodies against it. These antibodies neutralize the virus, preventing its interaction with ACE2 and reducing the likelihood of infection. This is the desired mechanism, which ideally should provide at least temporary protection after vaccination.

However, a problem arises when these same anti-S antibodies bind to the S proteins expressed by the body’s cells instead of simply neutralizing the virus. They can consequently attack the very cells that produce the S protein, as in the case of cardiac cells. This phenomenon represents one of the primary issues with this vaccination strategy and constitutes a serious conceptual flaw in the design of these vaccines.

In the lower part of Figure 5, a possible pathogenetic role of idiotype anti-ACE2 antibodies is outlined. The hypothesis is that vaccination and COVID-19 complications give rise to anti-idiotypic antibodies whose spatial shape can lead to interactions with ACE2 molecules, especially in individuals with prolonged S protein synthesis. Others have suggested that anti-idiotypic antibodies, with a shape similar to the S protein, likely interact with ACE2 molecules and cause various symptoms [103]. This mechanism does not represent a pathological condition; it is a physiological phenomenon of immune self-regulation that helps balance the presence of antibodies and prevent excessive production of new immunoglobulins. It should also be considered that each antibody has idiotypes and that anti-idiotypes give rise to further generations of antibodies (Ab3, Ab4, etc.), thus generating a regulatory network. Some researchers consider exploiting this mechanism to create neutralizing antibodies against the influenza A virus [125].

However, in some circumstances, this balance can be altered, leading to dysregulation. This imbalance can manifest itself through overproduction of the anti-idiotype, where the Ab2 antibody not only attacks the primary antibody but also begins to interact with other biological structures, generating unexpected and potentially harmful effects. This is precisely the phenomenon we observed in our studies, which are part of a line of research pursued by various groups [20,71,107,108].

One possible scenario is the following: when the production of anti-S antibodies becomes excessive, which refers to a condition of immunological overload, the immune system responds by generating large quantities of anti-idiotype (Ab2) antibodies. These antibodies have the ability to bind to anti-S (Ab1) antibodies, which, in turn, recognize and bind to the S protein. Notably, Ab2 antibodies can assume a structural conformation similar to the S protein itself. This means that anti-idiotypes develop an unexpected ability: they can interact with the ACE2 receptor, the same receptor targeted by the viral S protein. This topic has been discussed extensively in other articles [24,126].

The fact that anti-ACE2 doses correlate with anti-S levels [46,127] supports this hypothesis. Other authors have observed a significant increase in anti-ACE2 after COVID-19 vaccination [20,128]. Finally, Collins et al. [108] showed that sequential SARS-CoV-2 mRNA vaccination of hACE2 transgenic mice can induce both anti-S and anti-ACE2 antibody responses, and the latter was gradually amplified with each boost. This finding is very important because it provides experimental confirmation of empirical observations in humans and demonstrates that vaccination is not only followed by antigen-specific antibodies, as is commonly believed, but also by a series of secondary antibodies, which had not been observed in the registration studies. If a pathogenic role for anti-ACE2 antibodies is confirmed, their measurement should be included among the key controls in future trials of these newly developed vaccines.

### 3.2. The Role of MAS1

MAS1 encodes a G-protein-coupled receptor that binds ang-(1–7), with anti-inflammatory, antifibrotic, and antiangiogenic effects. This receptor is an integral part of the protective arm of the RAS, often referred to as the ACE2/ang-(1–7)/MAS1 axis. This axis counterbalances the effects of the classical ACE/angiotensin II/AT1 receptor pathway, known to promote vasoconstriction, inflammation, fibrosis, and hypertension [129]. The crucial role of ang-(1–7) in inflammation is evidenced by its concentration-dependent inhibition of the production and secretion of tumor necrosis factor alpha and interleukin-6 in LPS-induced macrophages. These inhibitory effects were reversed by A-779, a MAS1 receptor antagonist [130]. It has also been reported that, in a murine model of asthma, ang-(1–7) treatment exerted anti-inflammatory functions, mediated by the inhibition of the Src/EGFR/ERK1/2 signaling pathway [131].

Stimulation of MAS1 and/or its increased expression contributes to the maintenance of healthy vascular homeostasis by generating a gradual increase in prostacyclin, which reduces the risk of thrombosis through the increase in vasoprotective transcription factor Sirtuin 1 and the inhibition of platelet activation [132]. In laboratory models, ang-(1–7) showed neuroprotective effects, significantly reducing rotenone-induced oxidative damage in neuronal cells and increasing superoxide dismutase (SOD) and glutathione (GSH) [133,134]. In spontaneous hypertensive rats, ang-(1–7) injection exhibits hypotensive effects [135], and in Wistar rats, it counter-regulates the myocardial contractile response to acetylcholine [136]. These effects were blocked by the MAS receptor antagonist, A-779. Furthermore, ang-(1–7) increased the expression of MAS1 receptor and reduced the activation of NADPH oxidase, and both effects were completely eliminated by A-779 [137].

It has long been known that dysfunction of the ACE2/ang-(1–7)/MAS1 axis has profound repercussions on the central nervous system, particularly with regard to cardiovascular diseases, such as hypertension, chronic heart failure, and stroke, as well as neurological diseases [138]. Autoantibodies against MAS1 in patients with COVID-19 have been described by several authors [139]. In patients with PACS, characterized by a spectrum of neurological and cardiovascular symptoms, functionally active autoantibodies directed against MAS1 receptors have been detected [6]. Tested against rat cardiomyocytes, these antibodies exhibited a negative chronotropic effect [6].

The central role of the ACE2/ang-(1–7)/MAS1 axis is also suggested by the discovery of a rare variant of the MAS1 gene (c.446C > T p.(S149L) present exclusively in a patient with severe COVID-19, who died due to pneumonia and multiple thrombotic events [140]. Furthermore, an observational study analyzing the gene expression profiles of RAS receptors in hospitalized COVID-19 patients revealed an association between reduced MAS1 expression in elderly patients and in those who required oxygen therapy [141]. These results underline the importance of the ACE2/ang-(1–7)/MAS1 axis in regulating inflammation and suggest that targeting this pathway may have therapeutic potential.

It is difficult to explain the frequent increase in anti-MAS1 antibodies, as no homologies with viral or vaccine proteins have been described. A potential hypothesis is that of “epitope spreading” within the idiotypic network. Indeed, assuming that anti-ACE2 antibodies block enzyme function, it is conceivable that “similar” antibodies generated by the idiotype network and epitope spreading are also capable of interacting with MAS1. The binding site of ACE2 for angiotensin II should be very similar to the receptor binding site of MAS1, as the two proteins bind peptides that differ by only one amino acid, phenylalanine. A single antigen molecule can contain several epitopes recognized by different antibodies, and the same occurs for the variable chains of immunoglobulins, which are seen as new antigens. This process follows infections and complicates autoimmune diseases as tissue damage releases new antigens and related antibodies, leading to amplification of the immune reaction and disease persistence [113]. This phenomenon has also been invoked in the context of autoimmunity in long COVID [104].

### 3.3. Summary of the Immunological Dysregulation of RAS

In COVID-19 and post-vaccine pathologies, the RAS is at the center of immunological dysregulation of important regulatory systems, especially the cardiovascular and coagulation systems [101,139,141,142,143,144,145,146,147,148].

**Figure 6:**
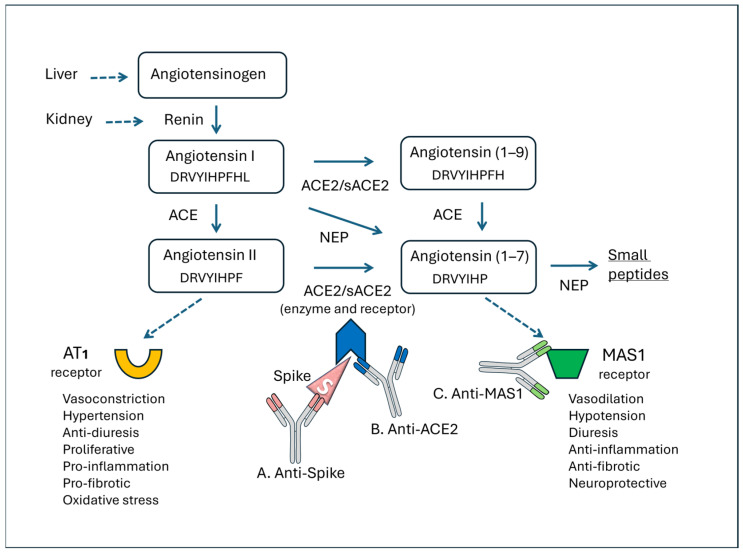
Scheme of the renin–angiotensin system and attack sites of anti-Spike (A), anti-ACE2 (B), and anti-MAS1 (C). For abbreviations, see the list at the end of the paper.

Figure 6 summarizes the function of the RAS and the main points where three different types of antibodies can act against the S protein, ACE2, and MAS1.

Renin, produced by the kidney in response to hypotension and hyponatremia, splits the angiotensinogen molecule at a leucine residue, generating the decapeptide angiotensin I, which is then converted into the biologically active product angiotensin II in the pulmonary capillaries. Angiotensin II mediates most of its physiological functions through the AT1 receptor, contributing to vascular homeostasis, but elevated levels are deleterious, causing vasoconstriction and oxidative stress [149]. ACE2 can cleave angiotensin I to generate the inactive peptide angiotensin-(1–9), which can then be converted to the heptapeptide ang-(1–7) via ACE or other peptidases. ACE2 can also directly metabolize angiotensin II to ang-(1–7). The latter peptide acts on the MAS1 receptor, promoting vasodilation, antiproliferation, and antihypertrophy. Therefore, two “axes” can be outlined in the RAS—ACE/Angiotensin II/AT1R and ACE2/ang-(1–7)/MAS1R—which have opposite functions. Ang-(1–7) is also generated via neprilysin [150], which cleaves angiotensin I. However, ang-(1–7) is further cleaved into smaller peptides [151]. ACE2 exists in a form bound to cell membranes and a soluble form (sACE2), both with enzymatic functions capable of cleaving angiotensin II and binding the S protein.

Anti-S antibodies (Figure 6A) can have both positive and negative effects on the RAS, as already outlined in the case of reactions associated with COVID-19 [24,126,152]. The positive effects are related to the elimination of the S protein, which could act agonistically or antagonistically on ACE2 functions. The agonistic actions of ACE2 involve receptor functions on membranes of platelets or leukocytes, which lead to cellular activation, platelet aggregation, and the release of inflammatory cytokines. On the other hand, the S protein may be bound to soluble ACE2 molecules [153] (sACE2), and in this case, these antibodies may lead to a depletion of plasma enzyme activity. The imbalance of ACE2 activity could be particularly severe in patients with a low baseline level of this receptor [154,155]. Downregulation of sACE2 can result in lung injury and vasoconstriction because conversion of angiotensin II to ang-(1–7) fails [153,156]. Furthermore, increased angiotensin II can have more extensive effects on tissues because it triggers multiple inflammatory responses through the ATR1 receptors, including the lungs [157].

With regard to ACE2 (Figure 6B), when these enzymatically active receptors become the target of an antibody attack, two potential outcomes can occur. First, if the antibodies block the receptor’s carboxypeptidase activity, this could lead to increased angiotensin II levels and subsequent activation of the AT1 receptor. This activation may lead to hypertension, pro-inflammatory effects, and endothelial dysfunction. Alternatively, if the antibodies are functionally active by stimulating the ACE2 receptor, they could mimic S protein activity and transmit cellular activation signals in endothelia, platelets, and leukocytes, thus contributing to pro-inflammatory cascades [24,25,88,158]. Another important pathophysiological connection is established between the RAS and the kinin system. As a carboxypeptidase, ACE2 cleaves many biological substrates in addition to angiotensin II to control vasodilation and vascular permeability [159]. In fact, ACE2 splits bradykinin into inactive peptides and therefore reduces kinin system activity, including vasodilation, increased endothelial permeability, and exudation. Dysregulation of bradykinin explains several mechanisms of inflammatory diseases and coagulation disorders. Various studies have highlighted the importance of the kinin system in COVID-19, describing a “kinin storm” responsible for uncontrolled phenomena, such as vasodilation, vascular permeability, and hypotension [160,161]. It is therefore conceivable that reducing ACE2 kininase activity could worsen symptoms of vaccine reactions, especially those of an inflammatory and painful nature.

Considering the studies reported, anti-MAS1 antibodies (Figure 6C) are relevant. These antibodies could block the MAS1 receptor [6] and prevent the beneficial action of ang-(1–7). The increase in ang-(1–7) observed in the majority of patients in our PACVS series may be related to this blockade and/or to the activation of compensatory mechanisms mediated by neprilysin, an enzyme that directly transforms angiotensin 1(1-19) into ang-(1–7) [150,162].

## 4. Clinical Implications

The complexity of PACVS immunological and metabolic disorders and the significant uncertainties regarding the mechanisms involved require a dynamic, adaptable clinical approach that constantly evolves based on advances in laboratory medicine and emerging therapies. In this section, we offer suggestions based on the underlying pathophysiological rationale and mechanisms to guide the diagnosis of this multifaceted syndrome and, where possible, to establish a rational therapeutic strategy, even in the absence of certainties in evidence-based medicine.

### 4.1. Diagnosis

A thorough family history is necessary for diagnosis because the most severe reactions often have a genetic basis, as with other vaccines [163], although genetics alone cannot be a cause of PACVS. Supportive or diagnostic tests are suggested based on clinical problems. Patients should be referred to the relevant acute services if they exhibit signs or symptoms that could be caused by an acute or life-threatening complication, including hypoxemia, signs of severe lung disease, cardiac chest pain, or multisystem inflammatory syndrome. People should be referred for psychiatric evaluation if they exhibit severe psychiatric symptoms or are at high risk of self-harm or suicide.

For cardiac evaluation, an ECG and echocardiogram should be performed. Cardiac magnetic resonance imaging with gadolinium contrast plays an important role in the noninvasive assessment of myocardial tissue in myocarditis and helps predict the prognosis of acute myocarditis based on specific phenotypic characteristics [164]. Measurement of troponin allows for the early diagnosis of myocardial infarction and post-vaccination myocarditis [165,166]. Troponin measurement is a valid marker of cardiac damage and may also be informative in autopsy, provided it is performed within 48 h of death [167]. For people with postural symptoms, such as palpitations or dizziness when standing, blood pressure and heart rate while lying down and standing should be recorded. High-resolution CT/CT chest scans could be performed if needed to evaluate for lung alterations.

If clinically indicated, blood tests should be performed, including a complete blood count, renal and liver function tests, analysis of key inflammatory markers, assessment of oxidative stress, and D-dimer, to monitor thrombotic risk. Useful laboratory markers are outlined in the following sections.

#### 4.1.1. Lymphocyte Populations

The relevance of lymphocyte population testing can vary greatly depending on the post-vaccination phase and the type of side effect. In the acute phase (reactions occurring in the first two weeks), testing is useful only if the patient has significant systemic reactions and an acute inflammatory or immune-mediated syndrome is suspected. If symptoms (asthenia, low-grade fever, myalgia, brain fog, or other viral infections) persist for more than 3 or 4 weeks, it may be useful to assess persistent CD4/CD8 imbalance, a reduction in B or T lymphocytes, or signs of adaptive immune dysfunction. Treg testing is important for evaluating potential autoimmune mechanisms. Persistent post-vaccination clinical alterations may occur even in the presence of normal antibody titers, suggesting the possible involvement of T-cell-mediated mechanisms or dysregulation of innate immunity.

#### 4.1.2. Anti-S and Anti-Nucleocapsid (N) Antibodies

Anti-S antibodies are indicated to assess the immune response to the vaccine ≥ 3–4 weeks after completion of the vaccination series and ≥ 2 weeks after the booster dose. They are useful in cases of suspected primary vaccine failure (COVID-19 disease contracted shortly after vaccination) [168], in immunocompromised patients, and to assess the possible cause of persistent post-vaccination adverse effects (indirect assessment of immune activation). We and others have noted a correlation between circulating antibodies and the severity of post-vaccination syndrome. Anti-nucleocapsid (N) antibodies are useful for distinguishing a vaccine response from previous or intercurrent natural infection, as these are not induced by vaccination. However, the interpretation of antibody levels has important limitations. First, progressive decay of anti-S antibodies over time is known, even in subjects with an initially adequate immune response; therefore, low antibody titers do not exclude functional immunological memory. Second, the antibody response does not fully reflect cellular immunity, which plays a central role in immunological protection and in modulating immune-mediated adverse events. In light of these considerations, the dosage of anti-S and anti-N antibodies should be interpreted in the clinical and temporal context and, in selected cases, integrated with the evaluation of cellular immunity (lymphocyte populations, CD4/CD8 ratio) and markers of systemic activation or inflammation.

#### 4.1.3. Inflammatory Cytokines

Inflammatory cytokines are key mediators of vaccination-induced activation of innate and adaptive immunity. Although the transient inflammatory response is a physiological mechanism necessary for the development of protective immunity, its persistence or deregulation can contribute to the onset and maintenance of systemic side effects. Inflammatory cytokine analysis should therefore be performed to assess the presence of a persistent state of immune activation, which may be associated with prolonged post-vaccination symptoms. Cytokines such as IL-6, TNF-α, IL-1β, and IL-8 are involved in modulating the systemic inflammatory response, as well as in influenza-like symptoms, asthenia, and musculoskeletal manifestations frequently reported after vaccination. Furthermore, growing evidence suggests that post-vaccination adverse events, especially when persistent, may reflect an unbalanced immune response rather than a simple humoral response, with possible involvement of innate immunity and cytokine signaling mechanisms [169]. IL-6 and IL-8 were elevated in 60% and 90% of PACVS-affected persons, respectively [12]. Recently, Patterson et al. observed statistically significant increases in IL-4, CCL3, CCL5, sCD40L, IL-8, and VEGF in patients with PACVS, while TNF-α and GM-CSF were downregulated [170]. A study on a group of 14 patients with PACVS found elevated levels of IL-10 (42.9% of cases), IL2 receptor (42.9%), IL-13 (28.6%), TNF-α (21.4%), and IL-12 (14.3%) [171]. Cytokine assessment, therefore, enables integration of serological and lymphocyte data, offering a more complete view of the immunological mechanisms underlying the observed side effects.

#### 4.1.4. Autoantibodies Against GPCRs

In parallel, evaluating anti-GPCR antibodies is advisable, particularly those targeting receptors involved in vascular and autonomic regulation (e.g., AT1R, β-adrenergic, muscarinic). These autoantibodies can act as functional agonists or antagonists, resulting in prolonged alterations in receptor signaling and dysautonomia symptoms, regardless of the presence of overt systemic inflammation. Emerging evidence suggests that the production of anti-GPCR antibodies may be triggered or amplified by intense immune activation, contributing to persistent clinical pictures characterized by endothelial dysfunction, autonomic dysregulation, and multisystem symptoms [58,172]. In this context, their evaluation allows us to explore immune-mediated mechanisms that cannot be detected through conventional inflammatory or serological parameters alone.

#### 4.1.5. Molecules of RAS and Related Autoantibodies

The evaluation of anti-ACE2 and anti-MAS1 antibodies was considered based on the hypothesis that these autoantibodies could functionally interfere with the RAS and contribute to persistent clinical manifestations. Measurement of these autoantibodies is not recommended as a routine screening test but may be considered selectively in the presence of persistent symptoms (>8–12 weeks) after COVID-19 vaccination, as well as signs and symptoms compatible with endothelial or vascular dysfunction, dysautonomia (tachycardia, orthostatic hypotension, exercise intolerance), or skin changes such as edema or ecchymosis. Therefore, their use is primarily in an exploratory and pathophysiological setting, rather than diagnostic, in patients with persistent and difficult-to-classify post-vaccination side effects. Antibodies against RAS-related receptors have been considered in selected cases to explore the possible involvement of autoimmune mechanisms and dysregulation of the RAS in subjects with persistent post-vaccination symptoms [29,173]. Italian authors reported a case series of 71 patients with CFS who presented with neurological symptoms, such as paresthesias and cognitive deficits, and dysautonomia, such as paroxysmal tachycardia, unrelated to exercise following SARS-CoV-2 vaccination. Of these patients, only nine (12%) were positive for anti-ACE2, resulting in no statistically significant difference compared to healthy donors. If these data are confirmed, it may suggest that anti-ACE2 agents could help distinguish PACVS from SFN.

#### 4.1.6. Ang-(1–7) Dosage

Plasma ang-(1–7) dosage provides an indirect indication of the balance between the two arms of the RAS. Reduced values suggest a prevalence of the ACE/Angiotensin II/AT1R pathway, associated with endothelial dysfunction, chronic inflammation, oxidative stress, and increased cardiovascular risk. Relatively high or balanced values indicate greater activity of the protective pathway, with compensatory effects on angiotensin II excess. The dosage may be useful in contexts of endothelial dysfunction, cardiovascular and metabolic pathologies, and chronic inflammatory conditions. Although not yet a routine biomarker, measuring ang-(1–7) can help guide RAS rebalancing strategies and support the use of ACE inhibitors or angiotensin receptor blockers. It is advisable to evaluate the impact of anti-inflammatory, metabolic, and lifestyle interventions (physical activity, insulin control, reduced oxidative stress) in research settings and monitor therapies targeting the MAS1 receptor or ang-(1–7) analogs. Limitations include high pre-analytical and analytical variability, low plasma concentrations, and difficulty with standardization. Interpretation should always be integrated with angiotensin II, ACE, ACE2, and the clinical context.

#### 4.1.7. Basophil Activation Test

A basophil activation test (BAT) may have instrumental value, but it is generally not useful for studying general immunological side effects following COVID-19 vaccination, except in very specific and selected cases where IgE-mediated allergy is suspected.

#### 4.1.8. Identification of Vaccine mRNA and S Protein

In the case of mRNA vaccines, PCR can be used to identify fragments of vaccine mRNA through primers designed for unique sequences of the vaccine construct (e.g., regions not present in the natural virus, such as specific UTRs or modifications of the S protein sequence) [89]. Vaccine-derived S proteins in the blood of COVID-19-vaccinated subjects can be measured via flow cytometry [170] and mass spectrometry, allowing them to be distinguished from virus-derived proteins [96,174]. In tissues, the presence of S can be identified via immunohistochemistry [25,175].

#### 4.1.9. Virus

In the context of acute infection, post-COVID-19, long COVID, or immune reactivation, it is often useful to evaluate other latent or persistent viruses that can influence the clinical course and immune response. Herpesviruses share the ability to establish latency and reactivate under conditions of immunological stress. HSV-1/HSV-2 are implicated in mucocutaneous reactivations, possibly contributing to systemic inflammation; VZV (HHV-3) is responsible for cases of reactivation (Herpes zoster) after viral infections or immunodisruption; and HHV-6/HHV-7 are associated with immune dysfunction, neuroinflammation, and chronic fatigue. EBV is one of the viruses most frequently implicated in post-COVID-19 reactivation, with possible associations with chronic fatigue, brain fog, myalgia, dysautonomia, polyclonal B lymphocyte activation, and increased cytokine production. EBV VCA IgM/IgG, EBNA IgG, and, in selected cases, EBV-DNA assessment is recommended. Cytomegalovirus (CMV) has a significant impact on immunosenescence, T cell responses, and the expansion of senescent CD8+ lymphocytes; it is also associated with worsening of inflammatory and vascular responses, which are relevant in patients with cardiovascular or immune comorbidities. Evaluation of CMV can be performed using specific IgG/IgM and, if indicated, CMV-DNA testing. Joint analysis of SARS-CoV-2 and latent viruses allows us to identify subclinical viral reactivations, explain the persistence of symptoms not explained by COVID-19 alone, assess the degree of immune dysfunction and chronic inflammation, and guide monitoring strategies, immunological support, and personalized follow-up. It is important to emphasize that PACVS should be considered a diagnostic hypothesis even if the patient’s clinical history includes a SARS-CoV-2 viral infection. Since these autoimmune diseases are caused, at least in part, by molecular mimicry, repeated exposure to similar antigens (the S protein) is likely to enhance responses, and the two causes (vaccine and virus) may overlap. This is important because it would be a mistake to rule out PACVS if the patient also experienced infection. Conversely, vaccination may worsen the course of PACS in some patients, particularly those vaccinated with Moderna (mRNA-1273) [176].

#### 4.1.10. Gut and Dysbiosis

The gut is a central immunological organ (GALT) and plays a key role in regulating systemic inflammation, immune tolerance, and metabolic balance. A dysbiotic and permeable gut can act as an amplifier of systemic inflammation and perpetuate symptoms even after an acute infectious event. Following viral infections (including SARS-CoV-2), immunological stress, or COVID-19 vaccination [177], gastrointestinal side effects can occur, including intestinal dysbiosis, increased intestinal permeability (“leaky gut”), and translocation of microbial antigens with chronic silent immune activation. In this context, it is interesting to note that dysbiosis has been detected in many patients with prolonged symptoms after COVID-19 (both vaccinated and unvaccinated) [178,179] and that the use of antibiotics improved their prognosis [180]. These mechanisms can contribute to persistent fatigue, gastrointestinal symptoms, neuroinflammation, endothelial dysfunction, and immune alterations. Intestinal permeability can be assessed via fecal or serum zonulin (an indirect marker of increased permeability) and measurement of plasma LPS or LBP (indicative of bacterial translocation). Intestinal inflammation can be assessed using fecal calprotectin, which distinguishes organic inflammation from functional inflammation, and fecal lactoferrin (if available). Finally, fecal microbiota analysis (NGS or targeted PCR) is very important, as it can reveal reduced biodiversity, an altered *Firmicutes*/*Bacteroidetes* ratio, and pathobiont overgrowth. This information is useful for personalized nutritional interventions, along with other techniques for assessing mucosal function, pancreatic insufficiency, the mucosal barrier, and fecal secretory IgA. Intestinal monitoring helps reduce chronic immune stimulation, support immune tolerance, improve responses to anti-inflammatory or immunomodulatory therapies, and guide interventions targeting diet, probiotics, prebiotics, polyphenols, and lifestyle.

#### 4.1.11. Oxidative Stress

Oxidative stress is a central pathogenic mechanism (activated by cytokines, angiotensin II, dysbiosis, and hypoxia) in conditions such as post-viral infection (including COVID-19), silent chronic inflammation, endothelial dysfunction, and RAS alterations [181,182]. This contributes to endothelial damage, mitochondrial dysfunction, and immunosenescence, perpetuating a vicious cycle of inflammation ↔ ROS. Evaluating oxidative stress markers is indicated in the presence of chronic fatigue, brain fog, myalgia, signs of endothelial or cardiovascular dysfunction, unexplained low-grade inflammation, leaky gut patterns, and bacterial translocation. Markers of oxidative damage include MDA or TBARS for lipid peroxidation, hydroperoxides (d-ROMs test) for global oxidative load, and measurement of 8-OHdG (urine), which highlights oxidative damage to DNA. Total antioxidant capacity can be assessed with a BAP test/CT scan and using the oxidant/antioxidant ratio, which is more informative than a single value. Elevated oxidative stress values are nonspecific, but they strengthen the hypothesis of persistent inflammatory activation and support endothelial and mitochondrial involvement. They are useful for monitoring the effectiveness of therapeutic interventions (nutrition, antioxidants, and targeted physical activity) and for assessing strategies to reduce upstream drivers (dysbiosis, elevated angiotensin II, and inflammation). Oxidative stress alone does not provide a diagnosis, but adds clinical value when integrated with inflammation, gut, endothelium, and RAS markers, especially during therapy and follow-up.

These biomarkers are not always present, as vaccine response is highly individual. Therefore, tests must be carefully and individually chosen.

As previously mentioned, distinguishing between PACVS and PACS is difficult because most people have had both viral infection and vaccination in their recent clinical history. However, criteria that can differentiate them exist, following recent scientific findings and the correct application of the WHO causality algorithm [66,67]. It is important to clarify that a causal relationship between vaccination and an adverse event is considered certain or at least probable when the adverse event develops within an appropriate time frame after vaccination, when there is biological or immunological plausibility, and when the relationship is supported by other similar cases in the scientific literature. In the case of PACVS, most of these requirements are met. It is also important to assess whether the individual has genetic susceptibilities to vaccinations, but these predisposing conditions should not be considered the “cause” of the disease, which depends on a combination of genetic and acquired factors, such as the vaccine trigger itself. The HLA system may also play a role as a genetic basis in this type of autoimmunity. According to Karami et al. [183], a peptide of the spike protein (Arg-Arg-Ala-Arg-Ser-Val-Ala-Ser) preferentially binds to various HLAs, including HLA-B*08:01 and HLA-B*07:02, which are typically associated with neurological autoimmune diseases. Others have noted a correlation between the presence of the haplotypes HLA-A*03:01 and HLA-A*29:02 and systemic side effects of COVID-19 vaccines, while HLA-B*08:01 appeared to play a protective role [184]. Obviously, the systematic identification of genetic conditions that predispose to PACVS, along the lines of an “adversomics” approach [33,185], is of great social and health significance.

### 4.2. Therapeutic Insights

Patients affected by organ diseases, which are already within the scope of established medical knowledge, should be treated with the most effective therapeutic approaches according to the literature and tailored to individual needs. For autoimmune reactions to vaccines and PACVS, there are no randomized trials demonstrating the efficacy of specific therapies, so decisions can only be based on pharmacological rationale, personal clinical experience, observational studies, and possibly long-term COVID guidelines (PACS). Based on the pathophysiological knowledge acquired to date and clinical experience, the most used drugs at the Institute of Biological Medicine in Milan (IMBIO), in order of priority, are glutathione, hesperidin + quercetin, ang-(1–7) in galenic formulation, nattokinase, bromelain, vitamin D, and antihistamines. We must reiterate that our experience, although conducted in accordance with ethical criteria and with the informed consent of patients, has not been tested on groups of patients compared with placebo or other drugs.

Supplementation with GSH may act as a protective factor at the cellular level against many types of oxidative stress, which in turn is a consequence of inflammatory phenomena and activation of NADPH oxidase. On the other hand, since cellular damage triggers inflammatory reactions, a vicious circle is generated that GSH helps to moderate by reducing the levels of inflammatory cytokines (such as IL-6 and IL-8) [186,187]. Furthermore, a good cytoplasmic GSH content hinders infections by opportunistic viruses, making the environment less hospitable for the pathogen [188]. The crucial importance of oxidative stress and the beneficial effects of GSH supplementation have been highlighted in both PACS and PACVS, in which there are various biochemical alterations of the same type [189,190,191]. Finally, the beneficial effect of GSH may be consistent with more recent evidence that transcriptomic profiles of some mRNA-vaccinated patients indicate cellular stress responses, mitochondrial dysfunction, and even oncogenesis [192].

Among the nutritional factors that may be useful in moderating the effects of autoimmunity are polyphenols, including hesperidin and quercetin, which, due to their antioxidant and anti-inflammatory properties, have already been indicated as potentially useful in COVID-19 disease [193,194,195,196]. The neuroprotective properties of flavonoids are also very important [197], as redox imbalance has been implicated in COVID-19-associated neuroinflammation, partly mediated by ACE2 depletion and Angiotensin II/AT1R/NADPH oxidase 2-mediated ROS generation, contributing to cognitive symptoms and neural apoptosis [49]. These two flavonoids have a high affinity for the receptor binding domain of the S protein and, according to molecular docking studies, are also able to bind to the ACE2 receptor [198,199]. In this way, the binding of hesperidin and/or quercetin physically hinders the attachment of the S protein to ACE2 and therefore the triggering of consequential inflammatory and thrombotic phenomena [24]. Clinical studies and meta-analyses also support quercetin supplementation in COVID-19 patients [200,201]. It is plausible that the same type of inhibition may occur with regard to the binding of anti-idiotype autoantibodies that have a similarity to the S protein. Furthermore, these flavonoids possess activity against viral reactivations, which are very frequent in this type of patient, in particular those of the herpesvirus family [202,203,204]. Obviously, once a viral infection has been ascertained, specific drugs can be administered, if effective ones exist. Others have also suggested the use of vitamins and polyphenols as complementary nutraceutical support in PACVS [16].

Since patients with COVID-19 and PACVS have a significant dysregulation of the ACE2/ang-(1–7)/MAS1 axis [24,46,141] and auto-antibodies against angiotensin I receptors and ACE2 [205], a new suggestion for the treatment of patients with PACVS could be the administration of ang-(1–7). In addition, in patients with heart failure, an increase in ang-(1–7) compared to angiotensin II is a predictor of positive outcomes and survival [206]. There are no efficacy studies of Ang-(1–7) in PACVS, but this heptapeptide improves physical performance in athletes [207], and animal studies support its supplementation as an anti-inflammatory, antithrombotic, and neuroprotective agent [134,208,209,210,211]. These results provide important preclinical evidence for strategies aimed at promoting the beneficial ang-(1–7)/MAS1 axis. An observational study by an Italian group, including one of the authors (GDF), suggested the usefulness of a complementary treatment comprising ang-(1–7) and endocannabinoids in autoimmune and neurodegenerative diseases [212]. However, it should be noted that the use of ang-(1–7) in patients with blockade of the MAS1 receptor could be, at least in theory, ineffective or counterproductive from a pharmacological perspective.

Another substance of potential utility is methylene blue (MB), which has been used in patients with COVID-19 to reduce the inflammatory markers and improve the respiratory parameters [213]. In addition, MB can reduce the oxidative stress [214] and prevent the interaction between protein S and the ACE2 receptor [215,216]. To date, no studies have explored the use of methylene blue in PACVS or PACS; however, there is evidence of beneficial effects in septic shock without significant adverse effects [217,218]. We used MB infusion in some patients who had particularly severe presentations, with promising results.

The use of nattokinase as a potentially useful agent for degrading the S protein has been included in therapeutic protocols, as previously suggested by other authors [219,220]. Bromelain has been rationalized based on its purported activity as an inhibitor of the spike–ACE2 interaction [221].

When necessary, we use drug therapies such as H1 and H2 antihistamines (e.g., cetirizine and famotidine) to manage pruritus, abdominal pain, dyspepsia, dysphagia, and nausea. Antihistamines also stabilize cells and help reduce inflammatory exudates and pulmonary distress, even in COVID-19 [222]. In some cases, steroids can be used for short periods, always in combination with complementary therapies. Magnesium is considered essential, as it is almost always deficient in chronic inflammatory conditions; it also has anti-histaminic and anti-inflammatory effects.

To remove excess autoantibodies and inflammatory mediators, in particularly severe cases refractory to other treatments, therapeutic plasmapheresis could be considered [45,223]. Finally, a study has been initiated on the infusion of immunoglobulins in patients with SFN following SARS-CoV-2 vaccination, which in some cases has yielded promising and long-lasting results [17].

## 5. Limitations

It is important to acknowledge that our study has some limitations. The data we reported, and on which our hypotheses are based, originate from a small retrospective study. In addition, we considered patients only from a single clinic. The limitation is offset by our attempt to place our observations within the broader framework provided by other researchers in the field of PACVS. Although the association between some symptoms, especially skin-related ones, and increased levels of anti-ACE2 and MAS1 emerged from a small sample, it is statistically significant enough to constitute preliminary evidence that deserves further exploration in larger case series. This result cannot be explained by selection bias, as we consecutively included all patients presenting to our clinic during 2023 with symptoms compatible with PACVS [46]. The only exclusion criterion was a history of COVID-19. Importantly, a statistical association does not mean that antibodies are the cause of the syndrome; a definitive conclusion would require direct demonstration of functional antibodies and, possibly, testing for those receptors in transgenic animals.

In this article, we have presented our experience with patients exhibiting symptoms that may be related to the syndrome described in the literature as PACVS. However, it must be acknowledged that, given the current state of knowledge, this diagnosis is still provisional, lacking clear laboratory and instrumental correlates and partially overlapping with other diseases. We cannot rule out that some patients may be classified as having other diseases triggered by COVID-19 vaccines, such as cardiovascular or neurological conditions. As case studies and knowledge of laboratory markers accumulate, it will be possible to better distinguish PACVS from other diseases with partially overlapping symptoms.

The presence of anti-ACE2 and anti-MAS1 antibodies still has significant interpretative limitations and does not automatically imply a causal link with clinical symptoms. Due to ongoing uncertainties regarding the definition of PACVS in the literature, the limited number of cases, and the lack of a healthy control group for comparison, it is not possible to establish the sensitivity and specificity of the various antibodies as biomarkers, due to the observational nature of our study. Indeed, the analysis of autoantibodies must be interpreted in the clinical and temporal context, preferably in association with other markers of immune and vascular dysregulation. Our aim was to report our observations and to propose mechanistic hypotheses regarding the immunopathologic alterations of the RAS, which warrant further investigation in prospective studies with larger sample sizes.

Finally, our conceptual approach emphasized the importance of autoimmune mechanisms in PACVS. However, we cannot rule out that the persistence of symptoms after vaccination may be due, at least in some patients, to prolonged spike protein persistence in serum or tissues. The biodistribution, quantity, and persistence of the S antigen following vaccination are not yet fully understood; however, the presence of the S protein in the lymph nodes and blood of patients vaccinated with mRNA has been found up to 245 days after the second dose, with considerable individual variations [96,170,224]. This analysis is not routinely performed in our laboratory, primarily due to ethical and cost reasons.

## 6. Conclusions

There is a growing body of literature describing the causes and pathophysiological mechanisms of PACVS. As a multifaceted syndrome with varying characteristics across individuals and partial overlap with the consequences of viral infection, the identification of a single biomarker is challenging. However, there is already significant evidence that the combination of clinical history and laboratory and instrumental tests may enable a diagnosis with high probability. Genetic predisposition to vaccine reactions (for example, autoimmune or cardiovascular diseases) does not exclude, but rather reinforces, the probability that damage was caused by vaccine administration.

PACVS represents a new frontier in clinical medicine, requiring a rethinking of diagnostic and therapeutic strategies [51]. Adopting a personalized clinical approach, based on a detailed analysis of patients’ immune and inflammatory profiles, is essential for addressing this complex syndrome. With continued research and collaboration among specialists, it will be possible to refine therapeutic strategies and offer patients more effective, long-lasting solutions. Importantly, all proposals based on scientific rationale, personal experience, and observational studies should be gradually evaluated in controlled studies comparing patients treated with different or partially different approaches, with the aim of optimizing therapies and eliminating ineffective drugs.

As knowledge about this syndrome advances, it is becoming increasingly important to classify PACVS with an appropriate ICD-10 code, as suggested by others [10]. This will allow patients to be included in public healthcare systems and recognized by health insurance systems.

Looking ahead, the discovery of the molecular mechanisms underlying autoantibody-mediated damage may improve PACVS diagnosis through the development of validated biomarkers and, ultimately, support more effective treatment and follow-up for this severe disease.

It is our hope that this work will serve as a basis for constructive dialogue, encouraging collaboration among professionals to identify innovative solutions and correct any errors encountered in clinical practice. Only through collective commitment and knowledge sharing can these new challenges be successfully addressed.

## Figures and Tables

**Table 1:** Signs and symptoms of PACVS.

Localization	Signs and Symptoms
Skin	Itching, urticaria, erythema, eczema, appearance of ruby angiomas, ecchymosis, petechiae
Respiratory system	Breathing difficulties even with small efforts, bronchospasm, asthma, and pulmonary fibrosis
Gastrointestinal system	Abdominal pain, diarrhea, nausea, bowel changes, dysbiosis, and microbiota alterations
Cardiovascular	Hypotension, hypertension, syncope, tachycardia from changes in position from supine to upright (POTS), extrasystoles, sudden transition from bradycardia to tachycardia
Central nervous system	Brain fog, amnesia, and difficulty concentrating, recurrent headaches, headache with aura, visual fog phenomenon, visual disturbances, accelerated progressive cognitive decline
Peripheral nervous system	Paresthesia of the peripheral limbs, neuropathic pain
Muscular and skeletal system	Osteomuscular pain
General	Asthenia, internal burning sensations

## Data Availability

The data presented in this study are available in aggregated form on request from the corresponding authors due to privacy reasons.

## Supplementary Materials

The following supporting information can be downloaded at: https://www.mdpi.com/article/10.3390/vaccines14040354/s1, Figure S1: Photo of the skin with severe itchy erythema accompanied by widespread burning sensations of the skin and subcutaneous tissue. Images are published with the written patient’s consent. Figure S2: Antibody panel of a patient with PACVS syndrome. Laboratory data are published with the written patient’s consent.

## Abbreviations

The following abbreviations are used in this manuscript:

ACE2	Angiotensin-converting enzyme 2
ANA	Antinuclear antibody
ANCA	Anti-neutrophil cytoplasmic antibody
Ang-(1–7)	Angiotensin-(1–7)
AT1R	Angiotensin II type 1 receptor
BAT	Basophil activation test
CFS	Chronic fatigue syndrome
CMV	Cytomegalovirus
CXCR3	C-X-C motif chemokine receptor 3
EBNA	Epstein–Barr nuclear antigen
EBV	Epstein–Barr virus
ENA	Extractable antinuclear antibody
ETAR	Endothelin A receptor
FGFR3	Fibroblast growth factor receptor 3
GALT	Gut-associated lymphoid tissue
GPCRs	G-protein-coupled receptor
GSH	Glutathione (reduced)
LNP	Lipid nanoparticle
HHV	Human herpes virus
HLA	Human leukocyte antigen
HSV	Herpes simplex virus
LBP	Lipopolysaccharide binding protein
LPS	Lipopolysaccharide
MAS1	Mas-related GPCR type 1
MTHFR	Methylenetetrahydrofolate reductase
N protein	Nucleocapsid protein
NADPH	Nicotine adenine dinucleotide phosphate (reduced)
NEP	Neprilysin
8-OHdG	8-hydroxy-2′-deoxyguanosine
PACS	Post-acute COVID-19 syndrome
PACVS	Post-acute COVID-19 vaccination syndrome
PCR	Polymerase chain reaction
POTS	Postural orthostatic tachycardia syndrome
PF4	Platelet factor 4
RAS	Renin–angiotensin system
ROS	Reactive oxygen species
SFN	Small fiber neuropathy
S protein	Spike protein
SOD	Superoxide dismutase
Stab1	Stabilin-1
Treg	Regulatory T cell
VCA	Virus capsid antigen
VZV	Varicella Zoster virus
