# Supplementary material for: Autoimmune Features of Post-COVID-19 Vaccination Syndrome and Their Impacts on the Renin–Angiotensin System
Source: Vaccines (Basel). 2026 Apr 16;14(4):354. doi: 10.3390/vaccines14040354 (PMC13120093; doi:10.3390/vaccines14040354)
Supplement: Supplementary file 1 [file vaccines-14-00354-s001.zip › vaccines-4235405-supplementary.pdf]

vaccines

MDPI

Review

Autoimmune Features of Post-COVID-19 Vaccination Syndrome and Their Impacts on the Renin-Angiotensin System

Supplementary information

This case is a 55-year-old woman who had always been in good health, except for mild anemia treated with iron supplementation, until the onset of post-COVID-19 vaccination illness. Vaccinated on March 22, 2021, with the viral vector vaccine (AstraZeneca), she initially experienced arthromyalgia and low-grade fever, which resolved with paracetamol. After three days, erythema appeared on the face and trunk. In the following weeks, she experienced worsening of widespread erythema, skin oedemas, itching, widespread arthralgia, and widespread burning paraesthesia. Over the years, the patient complained of recurrent hypertensive crises. These symptoms persist, with alternating severity, documented by repeated medical visits and sometimes even visits to the emergency room (Figure S1).

The patient is undergoing antihistamine therapy (cetirizine dihydrochloride) with partial improvement, while steroid therapy is ineffective. During a rheumatologist visit on August 18, 2021, the burning pain that occurred after vaccination was diagnosed as "*stinging dermographism reactive to an insect bite, allergy to something, vaccine not excluded*." Dermographism is a form of chronic inducible urticaria that occurs within minutes of the skin being stroked or scratched. However, no insect bites or their sequelae were found, and subsequent tests revealed no allergies. The adverse effects appeared a few days after vaccination and before first infection with SARS-CoV-2, which occurred and was documented by PCR on December 24, 2021.

Figure S2 shows the antibody panel we used. It can be seen that in this patient, most of the antibodies were negative (numbers in green); anti-α-1-adrenergic receptor and anti-β-2-adrenergic receptor were just above normal (numbers in orange), while the anti-MAS1 level was 94.5 U/ml, well above the threshold of pathology (number in red).

ANA, ENA, ANCA antibody assays yielded negative results.

Genetic analysis performed for further diagnostic purposes has documented the presence of both the C677T and A1298C mutations/polymorphisms in the methylenetetrahydrofolate reductase (MTHFR) gene in the heterozygous state.

Recent tests conducted in our laboratory (February 2026) revealed a high level of anti-SARS-CoV-2 S-RBD IgG antibodies (1,280.00 BAU/ml, when the positivity threshold is 4.33 BAU/ml) and of anti-SARS-CoV2 Nucleocapsid IgG and IgM antibodies (154.40 BAU/ml, when the positivity threshold is 1.10 BAU/ml).

Academic Editor: Loredana Frasca

Received: 17 March 2026
Revised: 13 April 2026
Accepted: 14 April 2026
Published: 16 April 2026

Copyright: © 2026 by the author. Licensee MDPI, Basel, Switzerland. This article is an open access article distributed under the terms and conditions of the Creative Commons Attribution (CC BY) license.

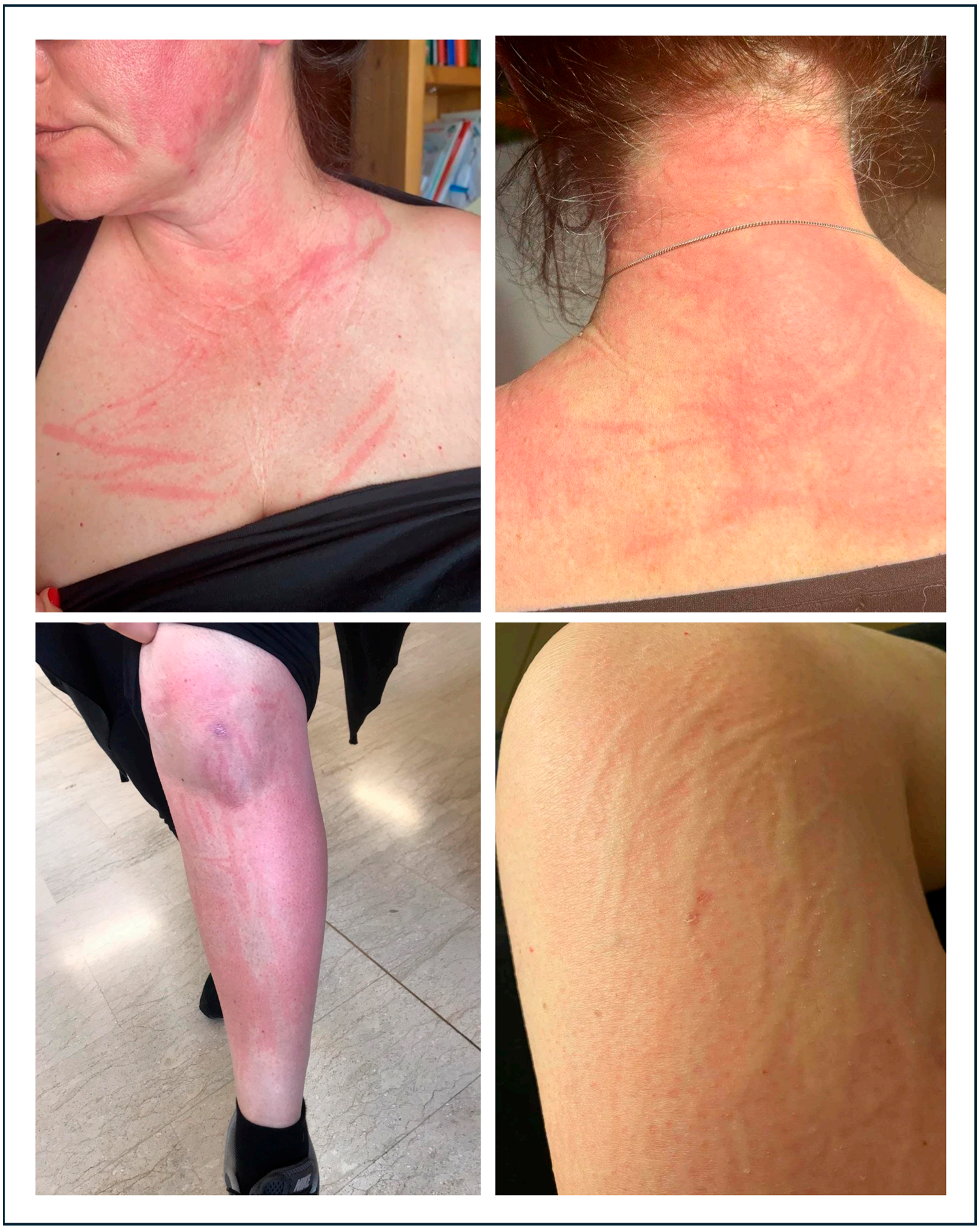

Figure S1. Photo of the skin with severe itchy erythema accompanied by widespread burning sensations of the skin and subcutaneous tissue. Images are published with the written patient's consent.

https://doi.org/10.3390/vaccines14040354

sample:	F[illegible]
date of birth:	1[illegible]
sample date:	07.01.2026
date of receiving:	08.01.2026
ordered by:	IMBIOLAB
method:	ELISA

RESULT REPORT

Parameter	Cut off	Units/ml
anti AT1R Antibodies	<10.0 U/ml: negative<br>10.0 – 17.0 U/ml: at risk<br>> 17.0 U/ml: positive	7,9
anti ETAR Antibodies	<10.0 U/ml: negative<br>10.0 – 17.0 U/ml: at risk<br>> 17.0 U/ml: positive	8,8
anti α-1-adrenergic Receptor Antibodies	<7.0 U/ml: negative<br>7.0 – 11.0 U/ml: at risk<br>> 11.0 U/ml: positive	*10,1*
anti α-2-adrenergic Receptor Antibodies	<15.0 U/ml: negative<br>> 15.0 U/ml: positive	9,6
anti β-1-adrenergic Receptor Antibodies	<15.0 U/ml: negative<br>> 15.0 U/ml: positive	7,7
anti β-2-adrenergic Receptor Antibodies	<8.0 U/ml: negative<br>8.0 – 14.0 U/ml: at risk<br>> 14.0 U/ml: positive	*8,0*
anti-Muscarinic Cholinergic Receptor-1-Antibodies	<9.0 U/ml: negative<br>> 9.0 U/ml: positive	3,7
anti-Muscarinic Cholinergic Receptor-2-Antibodies	<9.0 U/ml: negative<br>> 9.0 U/ml: positive	4,2
anti-Muscarinic Cholinergic Receptor-3-Antibodies	<6.0 U/ml: negative<br>6.0 – 10.0 U/ml: at risk<br>> 10.0 U/ml: positive	4,4
anti-Muscarinic Cholinergic Receptor-4-Antibodies	< 10.7 U/ml: negative<br>> 10.7 U/ml: positive	3,6
anti-Muscarinic Cholinergic Receptor-5-Antibodies	< 14.2 U/ml: negative<br>> 14.2 U/ml: positive	11,0
anti-FGF Receptor-3-Antibodies	<12.0 U/ml: negative<br>> 12.0 U/ml: positive	5,7
anti-TSHDS-IgM-Antibodies	<9.0 U/ml: negative<br>> 9.0 U/ml: positive	4,6
anti-ACE-2-Antibodies	<9.8 U/ml: negative<br>> 9.8 U/ml: positive	5,4
anti-MAS1-Receptor-Antibodies	<25.0 U/ml: negative<br>> 25.0 U/ml: positive	94,5
anti-PAR1-Antibodies	<4.2 U/ml: negative<br>> 4.2 U/ml: positive	2,7
anti-CXCR3-Antibodies	<40.0 U/ml: negative<br>> 40.0 U/ml: positive	10,8
anti-Stab1-Antibodies	<40.0 U/ml: negative<br>> 40.0 U/ml: positive	14,4

Figure S2. Antibody panel of a patient with PACVS syndrome. Laboratory data are published with the written patient's consent.

Disclaimer/Publisher's Note: The statements, opinions and data contained in all publications are solely those of the individual author(s) and contributor(s) and not of MDPI and/or the editor(s). MDPI and/or the editor(s) disclaim responsibility for any injury to people or property resulting from any ideas, methods, instructions or products referred to in the content.

https://doi.org/10.3390/vaccines14040354
